# Effects of Exogenous Phenolic Acids on Haustorium Induction of *Cistanche deserticola* Seeds Based on Host Metabolome Data

**DOI:** 10.3390/ijms26073300

**Published:** 2025-04-02

**Authors:** Shixin Tan, Xiuli He, Ru Feng, Liang Shen, Qingyun Pang, Rong Xu, Sai Liu, Changqing Xu

**Affiliations:** 1 State Key Laboratory for Quality Ensurance and Sustainable Use of Dao-di Herbs, Institute of Medicinal Plant Development, Chinese Academy of Medicinal Science, Peking Union Medicinal College, Beijing 100193, China; t1825285607@163.com (S.T.); ihexl1995@foxmail.com (X.H.); frcloudy@163.com (R.F.); p20001226@163.com (Q.P.); sliu@implad.ac.cn (S.L.); xuchangqingmail@sina.com (C.X.); 2 Natural History Museum of China, Beijing 100050, China; shenliang08@126.com

**Keywords:** *Cistanche deserticola*, *Haloxylon ammodendron*, root-parasitic plants, metabolomics, haustoria-inducing factors, medicinal plants

## Abstract

*Cistanche deserticola*, a holoparasitic plant widely used in traditional Chinese medicine, relies on chemical signals from its host plant, *Haloxylon ammodendron*, to initiate seed germination and haustorium induction. This study employed UPLC-MS/MS to analyze the root metabolites of *H. ammodendron*. The results showed that 11 substances such as phenolic acids, flavonoids, and alkaloids were mainly contained in the roots of *H. ammodendron*, among which phenolic acids accounted for the largest proportion, accounting for 18.00% in winter samples and 16.11% in autumn samples. Based on the reported exogenous substances that promote haustorium induction in *C. deserticola* and the differential metabolites in *H. ammodendron* roots, we selected seven exogenous signaling substances: 2,6-dimethoxy-1,4-benzoquinone, resorcinol, ferulic acid, syringic acid, vanillic acid, vanillin, and pelargonidin. Through concentration-gradient experiments (0.1–100 μM), we assessed their effects on haustorium induction in *C. deserticola* seeds. The results showed that among the seven substances, syringic acid, vanillic acid, and vanillin had the best impact on promoting the haustorium induction of *C. deserticola* seeds. Vanillic acid had the best impact at the concentration of 10 μmol/L, and the highest haustorium induction rate was 50.2%. There was no significant difference in the concentrations of vanillin and syringic acid. The results showed that phenolic acids in the host root system stimulated haustoria induction in *C. deserticola* seeds, with different substances requiring different optimal concentrations. This study not only identifies specific phenolic acids that enhance *C. deserticola* productivity but also establishes a chemical ecology framework for investigating host–parasite interactions in other root parasitic species.

## 1. Introduction

Some plants in nature cannot grow separately and rely on other plants for nutrients for their growth and development; such plants are called parasitic plants. Parasitic plants have evolved independently 12 or 13 times and account for about 1% of angiosperms [1]. Parasitic plants are characterized by their ability to invade the roots or stems of the host plant using a special structure known as a ‘haustorium’, to obtain water and a wide range of nutrients [2]. Chemical compounds of host origin that trigger haustorium induction are known as haustorium-inducing factors (HIFs). Given that haustorium induction frequently occurs in the roots of non-host species [3], it is hypothesized that these factors are common compounds widely distributed among plants.

Several chemicals have been reported as haustorium-inducing factors, including quinones, phenolics, anthocyanins, etc. [4]. Among them, 2,6-dimethoxy-1,4-benzoquinone (DMBQ), originally isolated from sorghum tissue extracts, is produced through the oxidation of syringic acid. Syringic acid is a phenolic compound probably derived from the mangiferin acid biosynthetic pathway and is an oxidative degradation product of lignin [5,6]. After identifying DMBQ, researchers found that many structural analogs of DMBQ can also induce haustorium induction in parasitic plants, such as syringic acid, caffeic acid, and ferulic acid, while phenolics, benzoquinones (BQs), and flavonoids can induce haustorium induction in vitro [7,8,9,10]. Available studies have shown that five classes of molecules, flavonoids, phenolic acids, quinones, cytokinins, and cyclohexene oxides, have been identified as active haustorium inducers [11].

*Cistanche deserticola* is a specialized root-parasitic plant, mainly on the roots of *Haloxylon ammodendron* and *Atriplex canescens*, and is a commonly used tonic traditional Chinese medicine (TCM) in China. *A. canescens* is a new host discovered in recent years [12], and the parasitic relationship between *H. ammodendron* and *C. deserticola* is more mature [13]. *C. deserticola* seeds need to be released from dormancy by low temperatures [7]. After the release of dormancy, *C. deserticola* seeds need to recognize the chemosensory substances secreted by the host root system to induce seed germination and haustorium induction to complete the parasitism process [14]. A previous study found that certain substances in the host root extract can affect the induction of haustorium in germinating *C. deserticola* seeds [15].

Plant secondary metabolites are compounds produced by plants to adapt to the growth environment, mainly including phenolic acids, terpenoids, alkaloids, etc. These compounds usually play a key role in the adaptation of plants to external stresses [16]. With the rapid development of metabolomics technology, metabolomics methods have been widely applied to the qualitative and quantitative analysis of plant metabolites [17]. Duan et al. showed that intercropping of leguminous crops has great potential to improve tea quality by studying the metabolic mechanisms of secondary metabolites in tea trees under the tea–soybean intercropping system [18]. Wang et al. clarified the metabolites of different medicinal parts of *Morus alba* L. through extensive targeted metabolome analysis, which provided a reference for the study of the medicinal and food values of *Morus alba* L. [19].

*C. deserticola* is usually inoculated in spring, and parasitic phenomena can be observed gradually in summer, autumn, and winter. To improve the inoculation rate of desert *C. deserticola* seeds and their yield, this experiment was conducted with *C. deserticola* seeds, using the rate of haustorium induction as an indicator, based on the changes in differential metabolites in the host plant’s different parasitic states and seasonal metabolome, combined with the exogenous haustorium-inducing factors reported in the literature [7,8,10,20,21,22,23,24]; the seven exogenous signaling compounds, namely DMBQ, resorcinol, ferulic acid, syringic acid, vanillic acid, vanillin, and pelargonidin were screened. The seven exogenous signaling substances were tested to determine the effects of different concentrations of these seven compounds on the induction of haustoria in the seeds of *C. deserticola*, to provide a theoretical basis for the study of the mechanism of haustorium induction in *C. deserticola* as well as the mechanism of parasitism.

## 2. Results

### 2.1. Analysis of Compound Metabolism Profiles of Haloxylon ammodendron Roots Parasitized by Cistanche deserticola and Non-Parasitized Haloxylon ammodendron Roots

We collected parasitized and non-parasitized *H. ammodendron* root materials from the Bencaocongrong Planting Base, Ningxia, China (Table 1), and seeds of *C. deserticola* were scattered around the root of *H. ammodendron* in the base. On 9 November 2019 (winter), we dug pits around *H. ammodendron* to observe whether they were parasitized and selected nine *C. deserticola*, nine parasitic *H. ammodendron*, and nine non-parasitic *H. ammodendron* as plant materials. On 30 August 2020 (autumn) and 15 July 2021 (summer), we followed the same steps to collect samples. The samples used in this experiment were identified by Professor Rong Xu of the Institute of Chinese Academy of Medicinal Science and Peking Union Medicinal College, and deposited in the germplasm resources of the Institute of Medicinal Plants, Chinese Academy of Medical Sciences Source Library.

UPLC-MS/MS analysis revealed that the metabolites of the host *H. ammodendron* could be classified into 11 distinct categories, with phenolic acids representing the predominant group. The QC sample mass spectrometry detection TIC overlap plot results show that the curve overlap of metabolite detection total ion flow is high and has good repeatability (Figure S1A–C). Principal Component Analysis (PCA) also showed good consistency among the groups, with significant differences between them (Figure S2A–C). In winter samples, a total of 389 metabolites were identified, including 70 phenolic compounds. Autumn samples exhibited the highest metabolite diversity, with 627 compounds detected, among which 101 were phenolic compounds. During the summer of 2021, this was only detected for phenolic acids.

**Figure 1:**
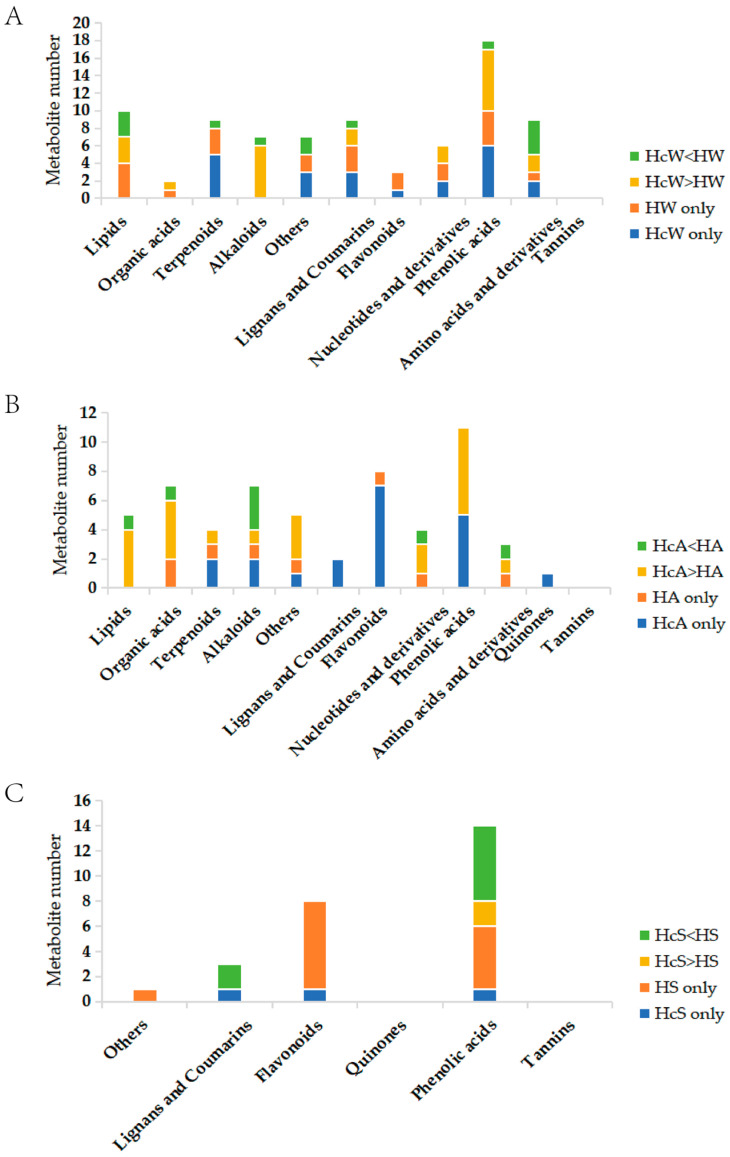
(**A**) Comparison of metabolites in HcW and HW roots screened using the OPLS model; (**B**) comparison of metabolites in HcA and HA roots screened using the OPLS model; (**C**) comparison of metabolites in HcS and HS roots screened using the OPLS model.

There may be differences in haustorium-inducing substances between *H. ammodendron* roots with parasitism of *C. deserticola* and *H. ammodendron* roots without parasitism. Therefore, this study first focused on the differences in secondary metabolites of *H. ammodendron* roots in the two states. Differential metabolites were mined by combining multivariate statistical analysis (VIP > 1 for OPLS-DA results) and univariate statistics (fold change ≥ 2 and fold change ≤ 0.5). In the comparative metabolomics analysis of parasitic and non-parasitic roots of *H. ammodendron*, winter samples showed 97 differentially expressed metabolites, with 38 significantly increased and 59 significantly decreased. The autumn samples showed 107 differentially expressed metabolites, with 46 significantly increased and 61 significantly decreased. The summer samples showed 50 differentially expressed metabolites, with 16 significantly increased and 34 significantly decreased. Among them, phenolic acids accounted for the largest proportion of differential metabolites, and the remaining differential metabolites were amino acids and their derivatives, other classes, alkaloids, lipids, nucleotides and their derivatives, flavonoids, and organic acids in turn (Figure 1). The further screened phenolic acid differential metabolite information is shown in Table 2, Tables S1 and S2 (Figures S3 and S4).

Among these differential metabolites, potential haustorium-inducing substances of *C. deserticola* may be present, yet the majority are likely secondary metabolites elicited in the host plants in response to *C. deserticola* parasitism. Haustorium-inducing factors are not expected to significantly differ between parasitic and nonparasitic roots of *H. ammodendron*, yet variations exist among distinct host plants, reflecting the varying probabilities of parasitism by *C. deserticola*. Consequently, this study refrained from verifying the haustorium-inducing potential of all differential metabolites, instead opting to screen them based on seasonal variations and literature-reported haustorium-inducing substances.

### 2.2. Metabolomic Comparative Analysis of H. ammodendron roots in Different Seasons Screening Possible Species of Seed Germination and Parasitic Signal Substances of C. deserticola

When conducting metabolite testing on samples from the winter of 2019 and the autumn of 2020, it was found that phenolic acid metabolites had the highest quantity and were most likely to induce haustorium activity. Therefore, when testing samples from the summer of 2021, emphasis was placed on selecting polyphenolic substances for detection.

The metabolite analysis results of *H. ammodendron* roots showed that the metabolites screened from *H. ammodendron* samples in three seasons contained a total of 50 metabolites with the same CAS number. Comparative analysis revealed that 28 of these 50 same metabolites were expressed higher in the parasitic roots than in the non-parasitic roots of *H. ammodendron*, among which salicylic acid, glucooxybenzoic acid, cimidahurinine, 5-(2-hydroxyethyl)-2-o-glucosylphenol, coniferoside were the most obvious, which may be related to *H. ammodendron* resistance. Seasonal metabolomic profiling revealed distinct seasonal variations among the three seasons, with 2 unique metabolites exclusively present in summer, 2 specifically identified in autumn, and 29 exclusively detected in winter (Table S3). Most of the metabolites in winter may be related to cold resistance, while *C. deserticola* generally does not germinate and parasitize in winter. The research team’s previous experiments measured that the root exudates of *H. ammodendron* collected in winter have little effect on seed germination and haustorium induction of *C. deserticola*, so we speculate that there are no haustorium-inducing substances in the substances unique to winter. However, this study chose pelargonidin as a negative control and presumed validation for the bioassay experiment.

The different target compounds were analyzed by comparing the metabolic components of roots in different seasons and parasitic states. We combined them with reported parasitic plant haustorium-inducing substances (Table 3) and further compared the metabolome data of *H. ammodendron* roots collected in different seasons, and screened out more than 20 phenolic acids and 12 flavonoids related to the germination of *C. deserticola* seeds and haustorium induction through seasonal and inter-group component differences.

**Figure 2:**
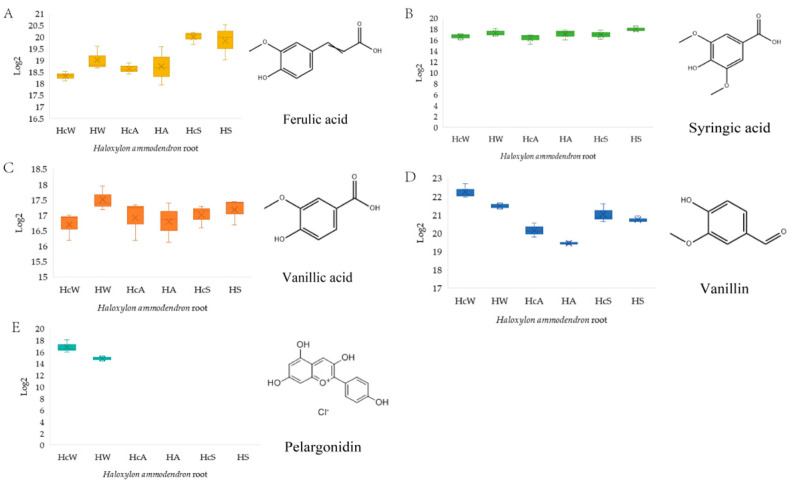
Content of five metabolites in *H. ammodendron* roots collected in different seasons. (**A**) Ferulic acid; (**B**) syringic acid; (**C**) vanillic acid; (**D**) vanillin; (**E**) pelargonidin.

Nearly 20 target compounds related to seed germination and haustorium induction of *C. deserticola* were analyzed by comparing the metabolic components of roots in different seasons and parasitic states. Combined with the reported haustorium-inducing factors of parasitic plants (Table 3), seven compounds were finally selected for bioassay experiments (Figure 2). Among them, DMBQ and resorcinol (RS) were not detected in the *H. ammodendron* metabolome, but they had obvious haustorial induction in other parasitic plants, as a control; pelargonidin (P) was only present in winter samples as a control. Ferulic acid (FA), syringic acid (SA), vanillic acid (VA), and vanillin (V), which were selected from the differential metabolites of phenolic acids and the reports in the literature, were selected as the main research objects. Five different concentrations (C1: 1000 μmol/L, C2: 100 μmol/L, C3: 10 μmol/L, C4: 1 μmol/L, C5: 0.1 μmol/L) were set to explore the induction effect on the haustorium of germinated *C. deserticola* seeds.

Vanillin concentrations exhibited a seasonal decline in non-parasitic roots (HA and HW) compared to parasitic counterparts (HcA and HcW) in autumn and winter, yet this pattern reversed in summer. In contrast, ferulic acid and syringic acid displayed an increasing trend in non-parasitic roots (HA and HW) relative to parasitic roots (HcA and HcW) during autumn and winter, with the trend reversing in summer. Vanillic acid levels in parasitic roots (HcW) were significantly elevated compared to non-parasitic roots (HF) in winter, yet this disparity diminished in summer. Pelargonidin was exclusively detected in winter samples of both non-parasitic (HW) and parasitic (HcW) roots.

### 2.3. Induction of Seed Haustoria in C. deserticola by Seven Exogenous Substances

**Figure 3:**
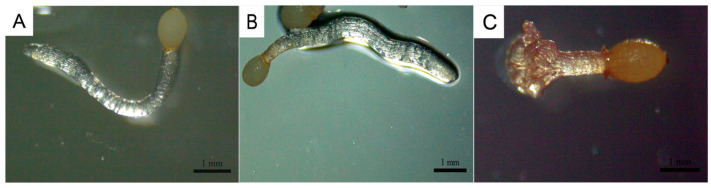
Microstructure of germ tubes of *C. deserticola* seeds after germination for 20 d. (**A**,**B**) No haustorium formed at the top of germ tube (scale bar: 1 mm); (**C**) the tip of the germ tube swells to form a haustorium (scale bar: 1 mm).

**Figure 4:**
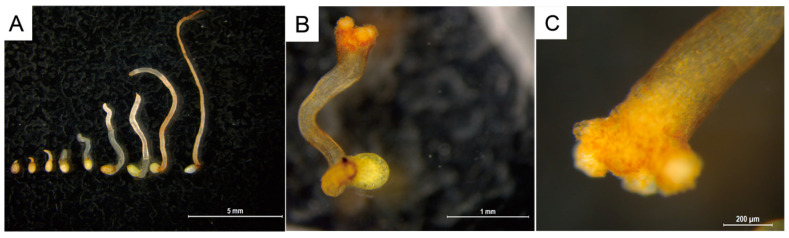
Micromorphology of germ tubes extending to atrophy and haustorium formation during germination of *C. deserticola* seeds (scale bar: 5 mm). (**A**) Germ tubes did not form haustorium growth process (scale bar: 1 mm); (**B**,**C**) the tip of the germ tube swells to form a haustorium (scale bar: 200 μm).

*C. deserticola* seeds belong to the spherical proembryo type. The embryo is simple in development and consists of dozens of cells. There is no differentiation of radicle, hypocotyl, and germ. Under suitable conditions, it germinates to produce “germ-like tubular organs”, also known as “germ tubes”. The shape and length of germ tubes vary greatly due to individual differences and the types and concentrations of sprouting stimulating substances. After germination, the germ tubes of different individual seeds vary in thickness, and their growth forms are upright, curved, or spiral [32]. Different *C. deserticola* seeds have obvious differences in the length of germ tubes after germination. Generally, the length of germ tubes is 0.5–4 mm, and the longest length of germ tubes can reach about 6 mm after 20 d of culture, which is more than 10 times the length of a seed kernel. Most germ tubes are white and slightly transparent, and some are light yellow or brown. A high-power microscope observed the microstructure of seed germ tubes of *C. deserticola*. The strip epidermal cells of germ tubes were elongated and longitudinally distributed, and the tip was swollen or appeared to be a sucker-like structure, which was called “haustorium” (Figure 3 and Figure 4). It could adhere to and invade the host root system and establish a parasitic relationship after connecting with the host plant vascular bundle. Under the condition of no host root system, the germ tube without haustorium will continue to elongate until it gradually shrinks after nutrient depletion in the seed endosperm [33] (Figure 4).

Through the analysis of the differential metabolites in the roots of parasitic and non-parasitic *H. ammodendron*, the results showed that phenolic acids were the main metabolites in *H. ammodendron* parasitized by *C. deserticola*. Combined with the literature and the previous research of the research group, DMBQ, resorcinol (RS), and pelargonidin (P) were selected as the control, and ferulic acid (FA), syringic acid (SA), vanillic acid (VA), vanillin (V) were selected from the phenolic acid differential metabolites and literature reports as the main research objects, and 5 different concentrations (C1: 1000 μmol/L, C2: 100 μmol/L, C3: 10 μmol/L, C4: 1 μmol/L, C5: 0.1 μmol/L) were used to explore the induction effect on the haustorium of germinated *C. deserticola* seeds. A total of 10 mg/L fluridone was added to each culture medium to promote seed germination.

**Figure 5:**
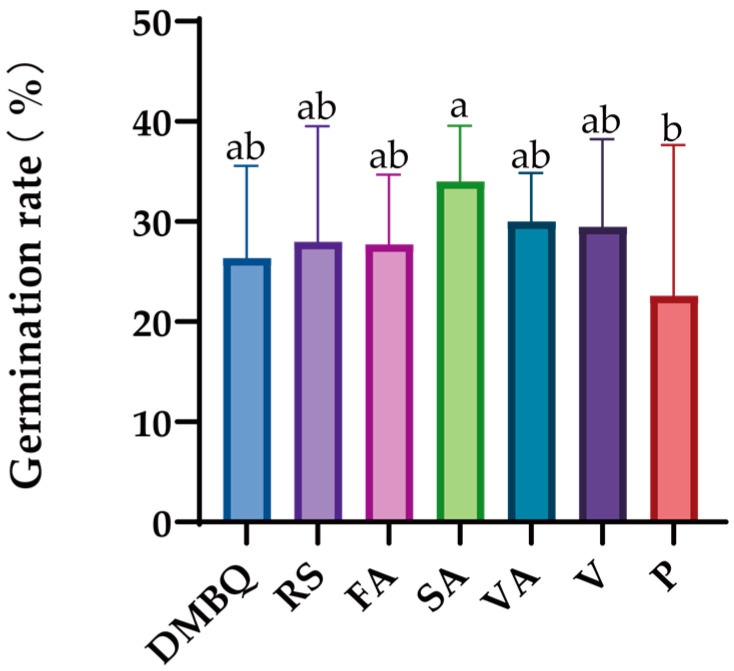
Effects of different substances on the germination induction rate of *C. deserticola*. Different letters indicate significant differences among samples according to Duncan’s test (*p* < 0.05).

The results showed that there was no significant difference in the effect of seven substances on the germination of *C. deserticola* seeds, but high concentrations (1000 μmol/L) of DMBQ, resorcinol, and pelargonidin inhibited the germination of *C. deserticola* seeds. The germination rate was higher at 0.1 μmol/L concentration, and the other four concentrations had no significant effect on the germination rate (Figure 5).

**Figure 6:**
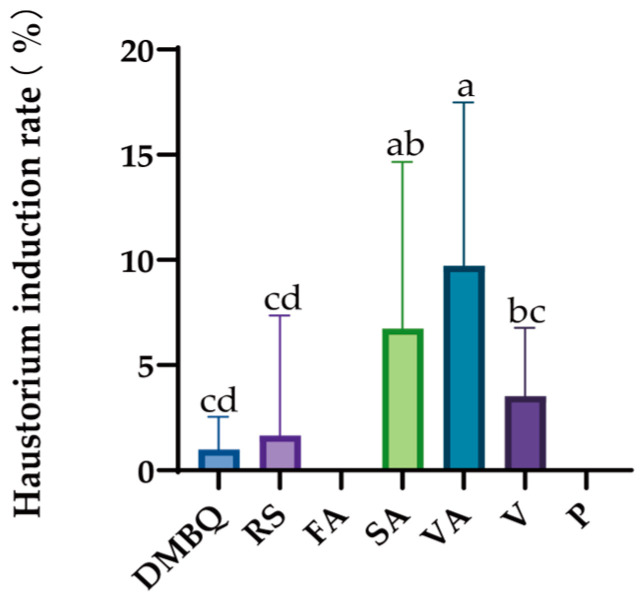
Effects of different substances on the haustorium induction rate of *C. deserticola*. Different letters indicate significant differences among samples according to Duncan’s test (*p* < 0.05).

Among the seven substances, DMBQ, Resorcinol, syringic acid, vanillic acid, and vanillin can promote haustorium induction in *C. deserticola* seeds. Vanillic acid has the best effect, followed by syringic acid and vanillin (Figure 6). Pelargonidin and ferulic acid had no haustorial induction effect.

**Figure 7:**
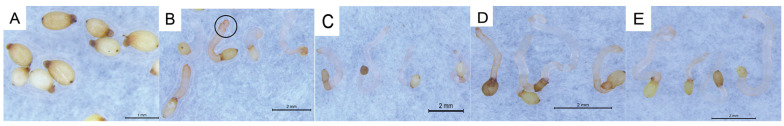
Induction of haustorium formation by DMBQ ((**A**): 1000 μmol/L(scale bar: 1 mm); (**B**): 100 μmol/L (scale bar: 2 mm); (**C**):10 μmol/L (scale bar: 2 mm); (**D**): 1 μmol/L (scale bar: 2 mm); (**E**): 0.1 μmol/L (scale bar: 2 mm)).

**Figure 8:**
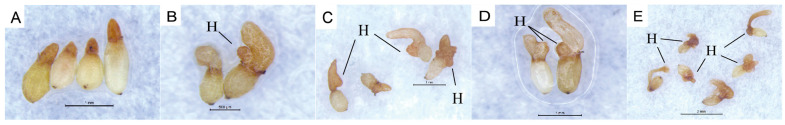
Induction of haustorium formation by syringic acid (H: haustorium) ((**A**): 1000 μmol/L (scale bar: 500 μm); (**B**): 100 μmol/L (scale bar: 1 mm); (**C**):10 μmol/L (scale bar: 1 mm); (**D**): 1 μmol/L; (**E**): 0.1 μmol/L (scale bar: 2 mm)).

**Figure 9:**
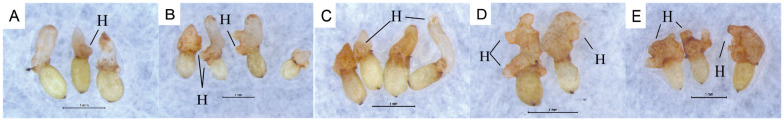
Induction of haustorium formation by vanillic acid (H: haustorium) ((**A**): 1000 μmol/L (scale bar: 1 mm); (**B**): 100μ μmol/L (scale bar: 1 mm); (**C**):10 μmol/L (scale bar: 1 mm); (**D**): 1 μmol/L (scale bar: 1 mm); (**E**): 0.1 μmol/L (scale bar: 1 mm)).

**Figure 10:**
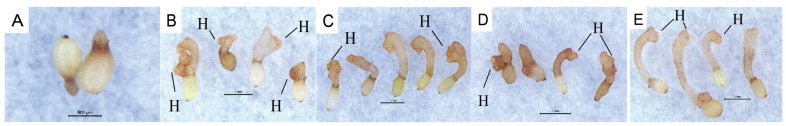
Induction of haustorium formation by vanillin (H: haustorium) ((**A**): 1000 μmol/L (scale bar: 500 μm); (**B**): 100 μmol/L (scale bar: 1 mm); (**C**):10 μmol/L (scale bar: 1 mm); (**D**): 1 μmol/L (scale bar: 1 mm); (**E**): 0.1 μmol/L (scale bar: 1 mm)).

It has been reported that DMBQ, which can promote the induction of haustoria of *C. deserticola*, only demonstrated a haustorium-like structure at 100 μmol/L in this study (Figure 7B), and only demonstrated bud tube growth at other concentrations (Figure 7). Among the three substances (syringic acid, vanillic acid, vanillin) with the best effect of haustorium induction, vanillic acid promoted the induction of haustorium at the optimal concentration of 10 μmol/L, and the highest haustorium induction rate was 50.2%. In vanillin and syringic acid, the concentration had no significant difference in promoting haustorium induction. The induction of haustorium in *C. deserticola* seeds can be observed at different concentrations of syringic acid, but the effect of inducing haustorium at high concentrations is not as good as that at low concentrations (Figure 8). In addition to vanillic acid, no haustorium was found at 1000 μmol/L, haustorium induction was found at other concentrations, and the number of seeds with haustorium induction was also more than that of other substances (Figure 9). In vanillin, no haustorium induction was found at 1000 μmol/L, and haustorium induction was found at other concentrations (Figure 10).

## 3. Discussion

*C. deserticola* is a holoparasitic plant. Nutrients and water are obtained from the host through haustoria [34], and the host releases signaling substances to induce haustorium induction, which are called haustorium-inducing factors. Many substances have been identified as haustorium-inducing factors, among which DMBQ can initiate the induction of haustorium tissues in many parasitic plants [35,36]; however, only haustorium-like structures were observed in this study, implying that different parasitic plants may have their specific haustorium-inducing factors.

In this study, different concentrations of different substances were found to have different effects on the induction of haustorium in *C. deserticola* seeds. Syringic acid, vanillic acid, and vanillin were all able to promote the induction of haustorium in *C. deserticola* seeds [37], in which the optimum concentration of vanillic acid to promote the induction of haustorium was 10 μmol/L, whereas there was no significant difference between vanillin and syringic acid in the concentration to promote the induction of haustorium. Therefore, the optimal concentrations of different substances to stimulate the induction of haustorium in *C. deserticola* seeds were different, suggesting that the induction of haustorium induction in natural production may be caused by the combined effect of many different signaling substances at different concentrations.

Studies on parasitic plants such as *Striga asiatica* and *Orobanche coerulescens* have shown that quinones, phenolics [38], cytokinins [4], and flavonoids [39] promote haustorium induction, while phenolics, benzoquinones, and flavonoids can induce haustorium induction in vitro [5]. In the present study, the metabolomic analysis of *H. ammodendron* parasitized by *C. deserticola* revealed eleven different substances, and we chose the phenolic acids, which accounted for the largest percentage, for testing, while others, such as amino acids and their derivatives, flavonoids, alkaloids, terpenoids, and esters, are yet to be investigated.

It is difficult to simulate the natural growth environment of *C. deserticola* in the laboratory for experiments on the haustorium-inducing factor, and this experiment was conducted only on seeds in Petri dishes. The results of this study will lay the foundation for the study of exogenous signaling substances to promote the induction of haustorium in germinating *C. deserticola* seeds in the soil matrix. Haustoria is the key to the establishment of a parasitic relationship between parasitic plants and their hosts, and the study of chemical signals in the process of haustorium induction in parasitic plants is not only of theoretical significance for the in-depth understanding of the relationship between parasitic plants and their hosts but also an important guide for improving the yield and quality of valuable parasitic plants.

## 4. Materials and Methods

### 4.1. Plant Materials and Test Reagents

According to the rule of the parasitic rate of *C. deserticola* previously investigated by the research group, we selected winter, autumn, and spring to obtain samples in ascending order of parasitic rate. The fleshy stems of *C. deserticola* and the roots of both parasitic and non-parasitic *H. ammodendron* were collected in November 2019, August 2020, and July 2021 from the *C. deserticola* planting base in Bencaocongrong Planting Base, Ningxia, China. After cutting the *C. deserticola* and *H. ammodendron* samples, all three materials from the same part of different plants were mixed as a sample, they were rinsed with purified water, cut into pieces, wrapped in tin foil, and placed in liquid nitrogen for rapid freezing. They were then transported to the Beijing laboratory on dry ice and stored in an ultra-low-temperature refrigerator at −80 °C. The seeds of *C. deserticola* were kept in a refrigerator at 4 °C. Chemical reagents such as fluridone, DMBQ, resorcinol, ferulic acid, syringic acid, vanillin, and pelargonidin were all of analytical purity.

### 4.2. Sample Preparation and Extraction

The freeze-dried leaf was crushed using a mixer mill (MM 400, Retsch, Shanghai China) with a zirconia bead for 1.5 min at 30 Hz. A total of 100 mg powder was weighted and extracted overnight at 4 °C with 0.6 mL 70% aqueous methanol. Following centrifugation at 10,000× *g* for 10 min, the extracts were absorbed (CNWBOND Carbon-GCB SPE Cartridge, 250 mg, 3 mL; ANPEL, Shanghai, China, www.anpel.com.cn, accessed on 15 September 2024) and filtrated (SCAA-104, 0.22 μm pore size; ANPEL, Shanghai, China, http://www.anpel.com.cn/, accessed on 15 September 2024) before UPLC-MS/MS analysis.

### 4.3. UPLC Conditions

The sample extracts were analyzed using a UPLC-ESI-MS/MS system (UPLC, Shim-pack UFLC SHIMADZU CBM30A system, www.shimadzu.com.cn/, accessed on 15 September 2024; MS, Applied Biosystems 4500 Q TRAP, Shanghai China, www.appliedbiosystems.com.cn/, accessed on 15 September 2024). The analytical conditions were as follows: UPLC: column, Waters ACQUITY UPLC HSS T3 C18 (1.8 µm, 2.1 mm × 100 mm); the mobile phase consisted of solvent A, pure water with 0.04% acetic acid, and solvent B, acetonitrile with 0.04% acetic acid. Sample measurements were performed with a gradient program that employed the starting conditions of 95% A and 5% B. Within 10 min, a linear gradient to 5% A, 95% B was programmed, and a composition of 5% A, 95% B was kept for 1 min. Subsequently, a composition of 95% A and 5.0% B was adjusted within 0.10 min and kept for 2.9 min. The column oven was set to 40 °C; the injection volume was 4 μL. The effluent was alternatively connected to an ESI-triple quadrupole-linear ion trap (QTRAP)-MS with both positive and negative ion modes.

### 4.4. ESI-Q TRAP-MS/MS

LIT and triple quadrupole (QQQ) scans were acquired on a triple quadrupole-linear ion trap mass spectrometer (Q TRAP), API 4500 Q TRAP UPLC/MS/MS System, equipped with an ESI Turbo Ion-Spray interface, operating in positive and negative ion mode and controlled by Analyst 1.6.3 software (AB Sciex, Shanghai China). The operating parameters of the ESI source are as follows: source temperature 550 °C; ion spray voltage (is) 5500 V (positive ion mode)/−4500 V (negative ion mode); the ion source gas I (GSI), gas II (GSII), and curtain gas (CUR) were set to 50, 60, and 30.0 psi, respectively, and the collision-induced ionization parameter was set to high. Instrument tuning and mass calibration were performed with 10 and 100 μmol/L polypropylene glycol solutions in QQQ and lit modes, respectively. QQQ scanning used MRM mode and collision gas (nitrogen) was set to medium. Through further DP and CE optimization, the DP and CE of each MRM ion pair were completed. A specific set of MRM transitions was monitored for each period as a function of the metabolites eluted in that period.

### 4.5. Principal Component Analysis (PCA)

Unsupervised PCA (principal component analysis) was performed by statistics function prcomp within R (www.r-project.org, accessed on 15 September 2024). The data were unit variance scaled before unsupervised PCA.

### 4.6. C. deserticolais Seed Treatment

The seeds were hulled, rinsed clean, soaked in water at 30 °C for 5 h, rinsed with 75% ethanol for 1 min, decanted with ethanol, rinsed with sterile water 3 times, then rinsed with 0.1% sodium hypochlorite solution for 3 min, fully shocked and shaken, decanted with 0.1% sodium hypochlorite solution, rinsed with sterile water 5 times, and then the surface of the seeds was dried with sterile filter paper.

### 4.7. Haustoria Induction Test

Firstly, the culture stock solution was configured. DMBQ (95% ethanol-assisted), resorcinol (ethanol- or water-assisted), ferulic acid (ethanol- or methanol-assisted), syringic acid (ethanol- or acetone-assisted), vanillic acid (ethanol-assisted), vanillin (methanol-assisted), pelargonidin (ethanol- or methanol-assisted), and fluridone (acetone-assisted) were configured into a 1000 μmol/L solution. All solutions were prepared and used on the same day. Except for fluridone, the seven solutions were diluted into five concentration gradients, and the five concentration gradients were 1000 μmol/L (C1), 100 μmol/L (C2), 10 μmol/L (C3), 1 μmol/L (C4), and 0.1 μmol/L (C5). Each culture solution was supplemented with 10 mg/L of fluridone, and 10 mg/L of fluridone was used as a control. There were three replicates in each group, 100 seeds were placed in each dish, 1.5 mL of culture medium was added, and the seeds were incubated in darkness at 25 °C for 25 d for observation. The experimental results were observed and photographed with a LEICA DFC450 body microscope. The rate of haustorium induction was counted, and the rate of haustorium induction = number of seeds forming haustoria/number of sprouted seeds × 100%.

## 5. Conclusions

Analysis of metabolomic data in this study revealed that many metabolites were increased in *C. deserticola*-parasitised *H. ammodendron* compared to non-parasitized *H. ammodendron*. These metabolites were phenolic acids, amino acids and their derivatives, flavonoids, alkaloids, terpenoids, and esters. Based on the literature and the analysis of differential metabolites, this study chose seven different phenolic acids to test and found that vanillic acid, vanillin, and syringic acid successfully induced haustorium induction, with different substances requiring different optimal concentrations. The induction rate of haustoria increases, which will effectively increase the yield of *C. deserticola* in actual production. This study lays the foundation for investigating the promotion of the formation of seed haustoria in germinating *C. deserticola* seeds by exogenous signaling substances in the soil matrix. This study only conducted experiments on phenolic acid substances, and the effects of other substances with high proportions in metabolites on the inhaler still need to be studied.

## Figures and Tables

**Table 1:** Information of plant materials.

	Simple ID	Species	Harvesting Time	Tissue
HcW	HcW1	*H. ammodendron*	9 November 2019	Root intruded by *C. deserticola*
	HcW2			
	HcW3			
HW	HW1			Root of healthy plant
	HW2			
	HW3			
HcA	HcA1		30 August 2020	Root intruded by *C. deserticola*
	HcA2			
	HcA3			
HA	HA1			Root of healthy plant
	HA2			
	HA3			
HcS	HcS1		15 July 2021	Root intruded by *C. deserticola*
	HcS2			
	HcS3			
HS	HS1			Root of healthy plant
	HS2			
	HS3			

**Table 2:** Phenolic acid differential metabolites detected by broad targeted metabolomics methods of HcW and HW.

Number	Compounds	Log2_FC	The Type of Regulation
1	Vanillin	1.23	up
2	Glucosyloxybenzoic acid	1.24	up
3	4-Hydroxybenzaldehyde	1.31	up
4	2,5-Dihydroxybenzoic acid	1.56	up
5	2,4-Dihydroxy benzoic acid	1.83	up
6	4-Hydroxybenzoic acid	1.88	up
7	Protocatechuic acid	1.93	up
8	vnilloylcaffeoyltartaric acid	2.14	up
9	Coniferyl alcohol	11.08	up
10	Salicylic acid	11.75	up
11	Vanillic acid	11.29	up
12	5-(2-Hydroxyethyl)-2-O-glucosylohenol	−1.45	down
13	feruloylmalic acid	−1.49	down
14	3-(4-Hydroxyphenyl)-propionic acid	−1.64	down
15	5′-Glucopyranosyloxyjasmanic acid	−1.71	down
16	Ferulic acid	−1.79	down
17	Syringic acid	−1.80	down
18	Echinacoside	−4.91	down
19	Syringin	−17.44	down

**Table 3:** Literature search of known haustorium-inducing substances in parasitic plants.

Plant	Haustorium-Inducing Factors
*Triphysaria versicolor*; *Striga hermonthica*; *Phtheirospermum japonicum*	DMBQ [7,21,25,26,27]
*Triphysaria versicolor*; *Striga hermonthica*	Syringic acid [10]
*Phtheirospermum japonicum*	Acetosyringone [5,10]
*Striga hermonthica*; *Triphysaria versicolor*; *Castilleja tenuiflora*	Vanillic acid [21,28,29]
*Agalinis*	Xenognosin A and B [30,31]
*Triphysaria versicolor*; *Phtheirospermum japonicum*; *Striga hermonthica*	Ferulic acid [10,21]

## Data Availability

All data in this study are available from the corresponding author upon reasonable request.

## Supplementary Materials

The following supporting information can be downloaded at: https://www.mdpi.com/article/10.3390/ijms26073300/s1.
