# Supplementary material for: Effects of Exogenous Phenolic Acids on Haustorium Induction of Cistanche deserticola Seeds Based on Host Metabolome Data
Source: Int J Mol Sci. 2025 Apr 2;26(7):3300. doi: 10.3390/ijms26073300 (PMC11989357; doi:10.3390/ijms26073300)
Supplement: Supplementary file 1 [file ijms-26-03300-s001.zip › Figure S3.pdf]

International Journal of
Molecular Sciences

MDPI

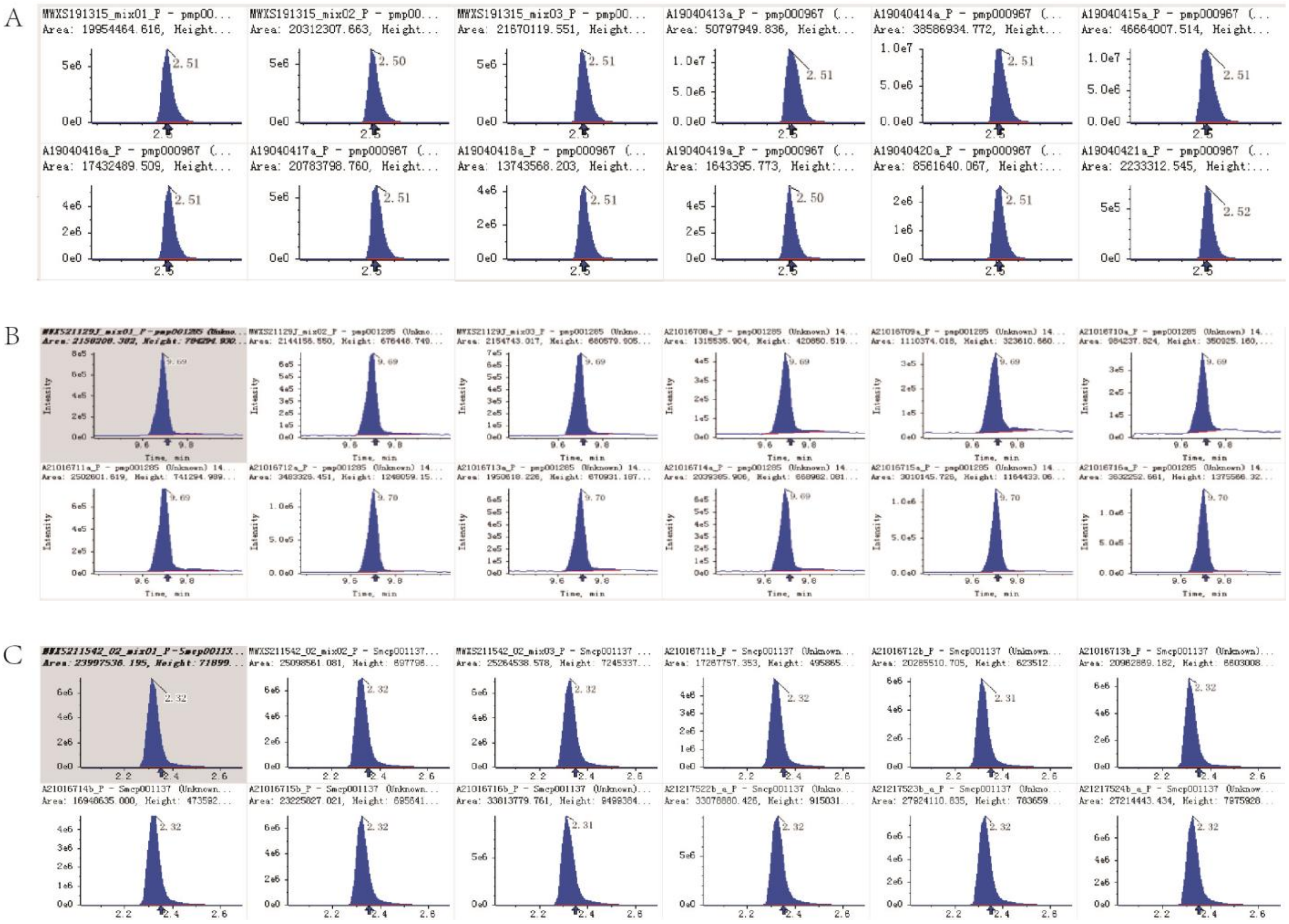

Figure S3. Mass spectrometry analysis of mixed sample total ion chromatogram. (A) 2019 sample; (B) 2020 sample; (C) 2021 sample.
