# Supplementary material for: Effects of Exogenous Phenolic Acids on Haustorium Induction of Cistanche deserticola Seeds Based on Host Metabolome Data
Source: Int J Mol Sci. 2025 Apr 2;26(7):3300. doi: 10.3390/ijms26073300 (PMC11989357; doi:10.3390/ijms26073300)
Supplement: Supplementary file 1 [file ijms-26-03300-s001.zip › Table S1.pdf]

International Journal of
Molecular Sciences

MDPI

Table S1. Phenolic acids differential metabolites detected by broad targeted metabolomics methods of HwA and HA.

Number	Compounds	Log2_FC	The type of regulation
1	Glucosyringic Acid	1.13	up
2	Methyl ferulate	1.23	up
3	Vanillic acid-4-O-glucoside	1.60	up
4	3-O-p-Coumaroylshikimic acid-O-glucoside	1.67	up
5	3,4,5-Trimethoxyphenyl-1-O-Glucoside	1.70	up
6	4-O-Glucosyl-sinapate	1.72	up
7	1-O-[(E)-p-Cumaroyl]-D-glucose	2.06	up
8	1-O-Glucosyl sinapate	2.09	up
9	Vnilloylcaffeoyltartaric acid	2.15	up
10	Trihydroxycinnamoylquinic acid	2.24	up
11	1-O-Gentisoyl-D-glucoside	2.55	up
12	Syringin	2.83	up
13	1-O-Feruloyl-D-Glucose	2.85	up
14	Disinapoyl glucoside	3.43	up
15	Rosmarinic acid-3'-O-glucoside	3.91	up
16	5'-Glucosyloxyjasmanic acid	4.80	up
17	Feruloylmalic acid	5.13	up
18	5-(2-Hydroxyethyl)-2-O-glucosylphenol	-1.05	down
19	Cimidahurinine	-1.08	down
20	3,4-Dihydroxybenzeneacetic acid	-1.34	down
21	Salicylic acid	-1.39	down
22	Tyrosol	-1.45	down
23	4-Hydroxybenzoic acid	-1.54	down
24	Coniferin	-1.61	down
25	1-O-[(E)-Caffeoyl]-D-glucose	-1.63	down
26	Cis-Coutaric acid	-2.40	down
