# Supplementary material for: Effects of Exogenous Phenolic Acids on Haustorium Induction of Cistanche deserticola Seeds Based on Host Metabolome Data
Source: Int J Mol Sci. 2025 Apr 2;26(7):3300. doi: 10.3390/ijms26073300 (PMC11989357; doi:10.3390/ijms26073300)
Supplement: Supplementary file 1 [file ijms-26-03300-s001.zip › Table S2.pdf]

International Journal of Molecular Sciences

MDPI

Table S2. Phenolic acids differential metabolites detected by broad targeted metabolomics methods of HcS and HS.

Number	Compounds	Log2_FC	The type of regulation
1	4-Hydroxybenzoic acid	1.36	up
2	Dihydrocaffeoylglucose	1.43	up
3	Vanillin acetate	1.48	up
4	4-O-(6'-O-Glucosylcaffeoyl)-3,4-dihydroxybenzoic acid	1.59	up
5	Grevilloside F	1.63	up
6	1-O-[(E)-Caffeoyl]-D-glucose	1.73	up
7	Vnilloyltartaric acid	1.75	up
8	6-O-Caffeoyl-D-glucose	1.96	up
9	Coniferin	2.00	up
10	3,4,5-Tricaffeoylquinic acid	2.10	up
11	Balajaponin B	2.50	up
12	Swerimilegenin I	2.63	up
13	Balajaponin E	3.55	up
14	Riboprine	3.89	up
15	Balajaponin C	10.14	up
16	Glucosyringic Acid	-1.04	down
17	1-O-Galloyl-4-O-p-Coumaroyl-β-D-glucose	-1.10	down
18	Ethyl cinnamate	-1.16	down
19	1-O-Glucosyl sinapate	-1.56	down
20	Arbutin	-1.62	down
21	3,4-dihydroxyphenylethanol-β-D-glucopyranoside	-1.76	down
22	Xylosyljasminoside B	-1.79	down
23	3-O-p-Coumaroylshikimic acid-O-glucoside	-1.91	down
24	Regaloside G	-2.18	down
25	3,6'-Disinapoylsucrose	-2.43	down
26	1-O-Feruloylquinic acid	-3.16	down
27	Shomaside E	-3.81	down
28	Feruloylmalic acid	-4.78	down
29	4-Hydroxyacetophenone	-9.65	down
30	Arillanin A	-12.17	down
31	Ferulic acid-4-O-glucoside	-15.19	down
