# Supplementary material for: Effects of Exogenous Phenolic Acids on Haustorium Induction of Cistanche deserticola Seeds Based on Host Metabolome Data
Source: Int J Mol Sci. 2025 Apr 2;26(7):3300. doi: 10.3390/ijms26073300 (PMC11989357; doi:10.3390/ijms26073300)
Supplement: Supplementary file 1 [file ijms-26-03300-s001.zip › Table S3.pdf]

International Journal of *Molecular Sciences*

MDPI

Table S3. Metabolites with differences in *H. ammodendron* roots in summer, autumn and winter.

Compounds	Season
Kaempferol-3-O-glucoside (Astragalin)	summer
Eriodictyol-8-C-glucoside	summer
Salidroside	autumn
3-Hydroxy-4-isopropylbenzylalcohol 3-glucoside	autumn
Pelargonidin	winter
Protocatechuic aldehyde	winter
3-methoxy-4-hydroxy-acetophenone	winter
Eudesmic acid(3,4,5-trimethoxybenzoic acid)	winter
Benzyl β-D-Glucopyranoside	winter
Salicin	winter
Isosalicylic acid O-glycoside	winter
Protocatechuic acid-4-glucoside	winter
4-Caffeoylquinic acid	winter
Chlorogenic acid	winter
3-Hydroxy-4-isopropylbenzylalcohol 3-glucoside-glucoside	winter
Daphnetin	winter
3-O-Galloyl-β-D-glucose	winter
O-Feruloyl 4-hydroxylcoumarin	winter
Esculin Hydrate	winter
Eupatilin	winter
Piperitol	winter
(8'R,7'S)-(-)-8-Hydroxy-α-conidendrin	winter
Isosinapic acid-hexoside	winter
Medioresinol	winter
Catechin gallate	winter
Epicatechin gallate	winter
Cistanoside I	winter
5,2'-Dihydroxy-7,8-dimethoxyflavone glycosides	winter
Cistanoside E	winter
Matairesinoside	winter
Terpineol monO-glucoside	winter
Syringaresinol-Hex	winter
Chrysoeriol-di-O-glucoside	winter
