# Supplementary figures and images for: Effects of Exogenous Phenolic Acids on Haustorium Induction of Cistanche deserticola Seeds Based on Host Metabolome Data
Source: Int J Mol Sci. 2025 Apr 2;26(7):3300. doi: 10.3390/ijms26073300 (PMC11989357; doi:10.3390/ijms26073300)

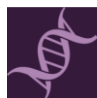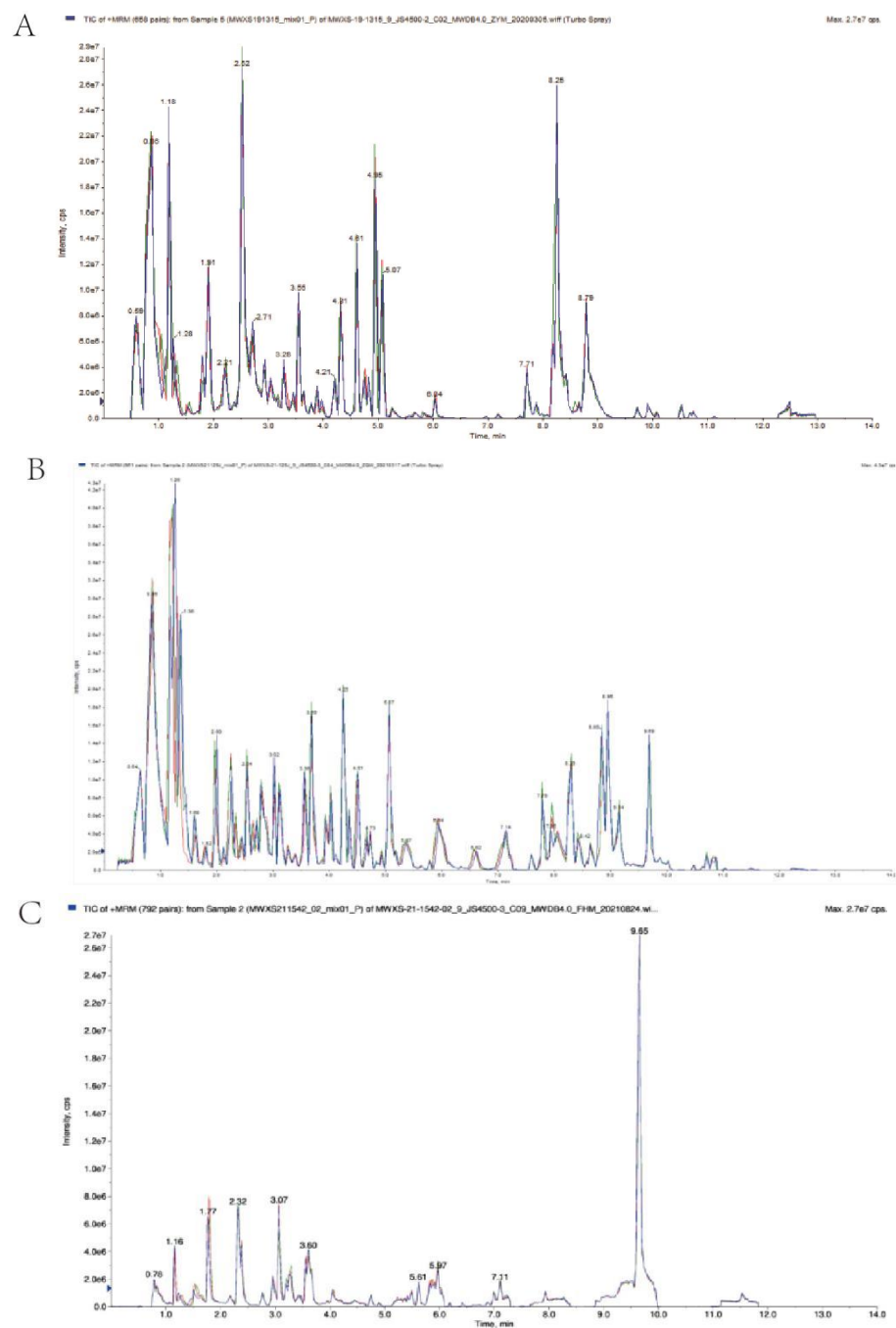

**Figure S1.** QC sample mass spectrometry detection TIC overlap map. (A) 2019 sample; (B) 2020 sample; (C) 2021 sample.

Supplement: Supplementary file 1 [file ijms-26-03300-s001.zip › Figure S1.pdf]

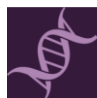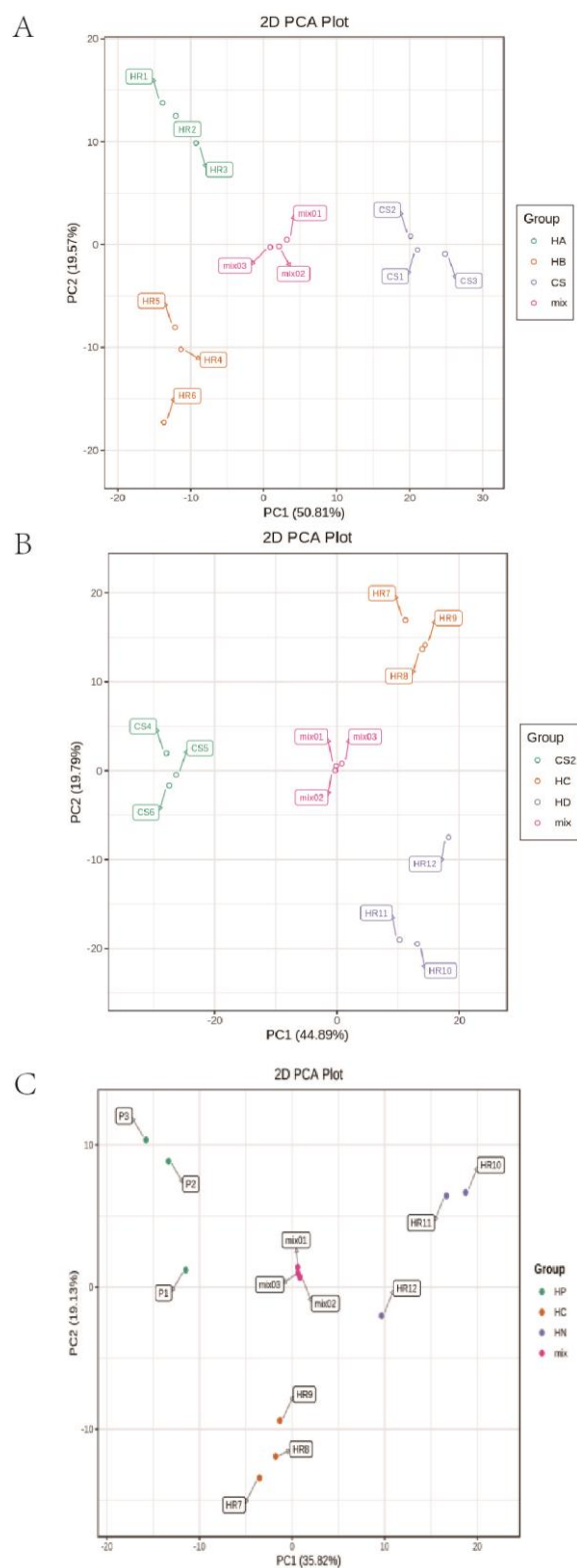

**Figure S2.** Principal component analysis (PCA). (A) 2019 sample; (B) 2020 sample; (C) 2021 sample.

Supplement: Supplementary file 1 [file ijms-26-03300-s001.zip › Figure S2.pdf]

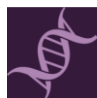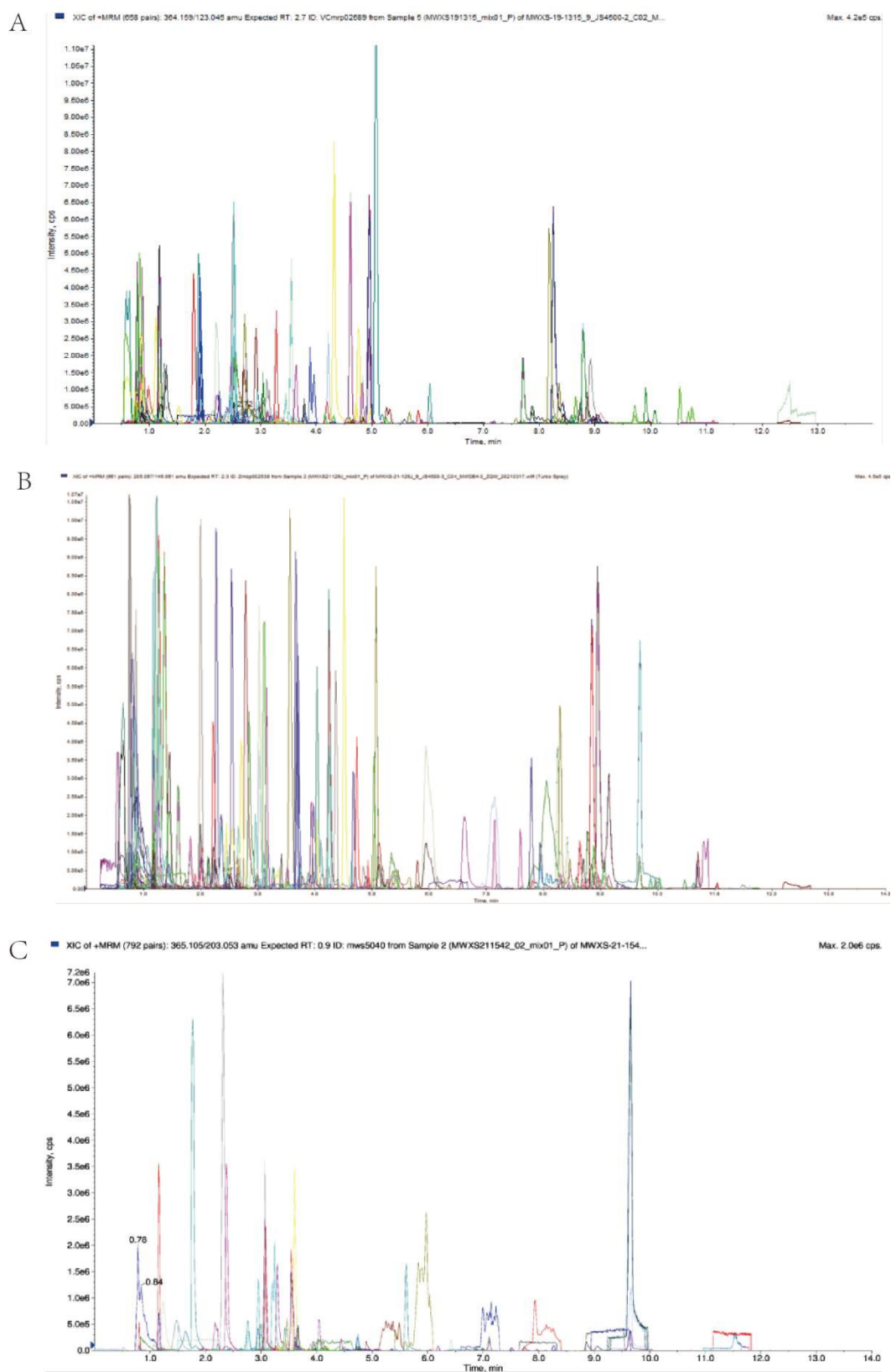

**Figure S4.** MRM metabolite detection multimodal graph. (A) 2019 sample; (B) 2020 sample; (C) 2021 sample.

Supplement: Supplementary file 1 [file ijms-26-03300-s001.zip › Figure S4.pdf]
